# Ivarmacitinib improves patient-reported outcomes across multiple domains in patients with active ankylosing spondylitis: a *post hoc* analysis of a phase II/III trial

**DOI:** 10.3389/fphar.2025.1710434

**Published:** 2025-11-20

**Authors:** Xiaoyan Xu, Hua Wei, Linchen Liu, Rong Yang, Qing Shi, Dandan Pang

**Affiliations:** 1 Department of Rheumatology, Zhongda Hospital, Southeast University, Nanjing, China; 2 Department of Rheumatology, Subei People’s Hospital of Jiangsu Province, Yangzhou, China

**Keywords:** ivarmacitinib, ankylosing spondylitis, patient-reported outcomes, high selective JAK inhibitor, phase II/III trial

## Abstract

**Background:**

Ivarmacitinib (SHR0302), a selective Janus kinase 1 inhibitor, has demonstrated substantial improvements in patients with active ankylosing spondylitis (AS). This *post hoc* analysis evaluated the effects of ivarmacitinib on various dimensions of patient-reported outcomes (PROs) in active AS patients, utilizing data from a phase II/III clinical trial (NCT04481139).

**Methods:**

Patients were assigned to receive either ivarmacitinib 4 mg (n = 187) or placebo (n = 186) daily for 12 weeks. Patients receiving placebo switched to ivarmacitinib thereafter until week 24. PROs included total back pain and night pain by visual analog scale (VAS), morning stiffness, Patient Global Assessment of Disease Activity (PtGA), AS Quality of Life (ASQol), 36-Item Short Form Health Survey (SF-36), Bath AS Disease Activity Index (BASDAI), and Bath AS Functional Index (BASFI).

**Results:**

Ivarmacitinib group showed significantly improvement in total back pain VAS (*P* < 0.001), night pain VAS (*P* < 0.001), morning stiffness (*P* < 0.001), PtGA (*P* < 0.001), ASQoL (*P* = 0.034), and BASDAI (*P* < 0.001) scores compared with placebo after 12 weeks of treatment. However, no significant between-group differences were observed for SF-36 physical scores (*P* = 0.216), mental (*P* = 0.105) component scores and BASFI score (*P* = 0.744) at week 12. By week 24, all PROs were continuously improved in the ivarmacitinib group; patients who switched from placebo to ivarmacitinib 4 mg achieved substantial improvements in all PROs.

**Conclusion:**

Ivarmacitinib significantly enhances multiple dimensions of PROs in active AS patients, supporting its utility in managing PROs in AS. Switching to ivarmacitinib also provides substantial benefits, this indicates that initiating ivarmacitinib treatment, even after an initial period of placebo leads to meaningful improvements in PROs.

## 1 Introduction

Ankylosing spondylitis (AS) is a chronic, inflammatory rheumatoid disease, which primarily affects the axial skeleton but may also involve other joints such as the hips and shoulders (Ebrahimiadib et al., 2021; Mauro et al., 2021). The main symptoms of AS are pain in the back and morning stiffness; meanwhile, AS also has enormous impacts on patients’ quality-of-life (QoL), daily functioning, and work productivity (Taurog et al., 2016; Zimba et al., 2024). The treatment goal for AS is to alleviate the symptoms and reduce skeletal destruction, thus improving the overall QoL in patients with AS (Choufani et al., 2024; Ward et al., 2019; Xie et al., 2020). As a result, the assessment of patient-reported outcomes (PROs) is vital in evaluating the treatment for AS.

Janus kinase (JAK) inhibitors target the JAK/Signal Transducer and Activator of Transcription (STAT) signaling pathway and further suppress the release of cytokines involved in the development and progression of autoimmune and autoinflammatory diseases (Kondo et al., 2021; Smith, 2015; Virtanen et al., 2024). Several clinical studies have reported that JAK inhibitors, such as upadacitinib, tofacitinib, and filgotinib, improved the treatment response in active AS (Deodhar et al., 2021; Deodhar et al., 2022; van der Heijde et al., 2018; van der Heijde et al., 2019). Moreover, JAK inhibitors could also improve diverse PROs reflecting pain, fatigue, and QoL in these patients (McInnes et al., 2022; Navarro-Compán et al., 2024; Navarro-Compán et al., 2022).

Ivarmacitinib (SHR0302) is a novel JAK inhibitor with a high affinity for JAK1 (Liu et al., 2025c). Previous clinical trials have demonstrated that ivarmacitinib could serve as a potential therapeutic agent for autoimmune and autoinflammatory diseases such as rheumatoid arthritis, atopic dermatitis, inflammatory bowel disease, etc. (Chen et al., 2022; Liu et al., 2025b; Zhao et al., 2025). Regarding AS, a recently published phase II/III clinical trial reported that ivarmacitinib 4 mg significantly p increased the proportion of patients achieving Assessment of Spondyloarthritis International Society 20% improvement (ASAS20) response at 12 weeks of treatment compared with placebo in patients with active AS (Liu et al., 2025c). The results of PROs are also briefly reported by the previous study (Liu et al., 2025c). However, in order to further support the application of ivarmacitinib in active AS, a more detailed presentation and interpretation of PROs after treatment is necessary.

Based on the previous phase II/III clinical trial, this *post hoc* study aimed to compare PROs between ivarmacitinib and placebo in patients with active AS.

## 2 Materials and methods

### 2.1 Study design

This *post hoc* analysis evaluated the effects of ivarmacitinib on various dimensions of PROs in active AS patients. The data were derived from a phase II/III clinical trial (NCT04481139), and the complete methodology has been published previously (Jiangsu HengRui Medicine Co, 2023). The study protocol was approved by the institutional review boards of all participating centers.

### 2.2 Patients

Detailed phase II/III clinical trial eligibility criteria were previously reported. Patients met the Modified New York criteria for AS and confirmed by radiographic evaluation of the sacroiliac joints. The active AS patients who received ivarmacitinib 4 mg (n = 187) daily for 24 weeks, or patients who received placebo for 12 weeks then switched to ivarmacitinib 4 mg for the subsequent 12 weeks (n = 186) in the phase II/III clinical trial, were included in this *post hoc* analysis.

### 2.3 Data collection

The clinical characteristics of AS patients were retrieved, which included age, sex, body mass index (BMI), smoking status, drinking status, disease duration, history of biological/JAK inhibitors, HLA-B27, C-reactive protein (CRP), and erythrocyte sedimentation rate (ESR). Besides, PROs were assessed, which included total back pain and night pain by visual analog scale (VAS), morning stiffness, Patient Global Assessment of Disease Activity (PtGA), AS Quality of Life (ASQoL), 36-Item Short Form Health Survey (SF-36), Bath Ankylosing Spondylitis Disease Activity Index (BASDAI), and Bath Ankylosing Spondylitis Functional Index (BASFI). Based on the PROs, the dimensions of pain, stiffness, overall disease activity, and the quality of life were assessed.

### 2.4 Statistics

The characteristics of sex, smoking status, drinking status, history of biological/JAK inhibitors, and HLA-B27 were shown as numbers (%), and the χ^2^ or Fisher’s exact test was utilized for comparisons. The characteristics of age, BMI, and disease duration were shown as the means with standard deviations (SDs), and the Student’s t-test was utilized for comparisons. CRP and ESR were shown as medians with interquartile ranges (IQRs), and the Wilcoxon rank sum test was employed for comparisons. PROs were shown using observed mean change from baseline, which was defined as the PROs value at each time point minus the PROs value at baseline, and were analyzed using Student’s t-test. Adjustments for multiplicity were not performed. SPSS 29.0 (IBM, United States) was used for statistical analysis. A two-sided *P* < 0.05 was considered statistically significant.

## 3 Results

### 3.1 Baseline characteristics between groups

Baseline characteristics were comparable between the ivarmacitinib and placebo groups, which was reported in the phase II/III clinical trial (Liu et al., 2025c). The mean age was 34.1 ± 10.0 years in the ivarmacitinib group and 33.5 ± 10.5 years in the placebo group. Mean disease durations were 9.5 ± 7.0 and 9.2 ± 6.8 years in the two groups, respectively. There were 61 (32.6%) patients in the ivarmacitinib group and 59 (31.7%) patients in the placebo group had a history of biological/JAK inhibitors. The median (IQR) levels of CRP at baseline were 7.8 (3.0–16.8) mg/L in the ivarmacitinib group and 7.0 (2.8–14.9) mg/L in the placebo group; meanwhile, those of ESR were 19.0 (8.0–34.0) and 16.0 (8.0–30.0) mg/L in the two groups, respectively (Table 1).

**TABLE 1:** Clinical characteristics.

Items	Placebo (N = 186)	Ivarmacitinib 4 mg (N = 187)	*P* value
Age (years), mean ± SD	33.5 ± 10.5	34.1 ± 10.0	0.583
Sex, n (%)			0.589
Male	146 (78.5)	151 (80.7)	
Female	40 (21.5)	36 (19.3)	
BMI (kg/m^2^), mean ± SD	24.1 ± 4.0	24.4 ± 4.3	0.626
Smoke status, n (%)			0.391
Never	114 (61.3)	110 (58.8)	
Former	7 (3.8)	13 (7.0)	
Current	65 (34.9)	64 (34.2)	
Drink status, n (%)			0.071
Never	133 (71.5)	132 (70.6)	
Former	9 (4.8)	2 (1.1)	
Current	44 (23.7)	53 (28.3)	
Disease duration (years), mean ± SD	9.2 ± 6.8	9.5 ± 7.0	0.666
Concomitant NSAID use, n (%)	158 (84.9)	155 (82.9)	0.596
History of biological/JAK inhibitors, n (%)			0.852
No	127 (68.3)	126 (67.4)	
Yes	59 (31.7)	61 (32.6)	
HLA-B27, n (%)			0.589
Negative	22 (11.8)	20 (10.7)	
Positive	161 (86.6)	166 (88.8)	
Not evaluated	3 (1.6)	1 (0.5)	
CRP (mg/L), median (IQR)	7.0 (2.8–14.9)	7.8 (3.0–16.8)	0.268
ESR (mg/L), median (IQR)	16.0 (8.0–30.0)	19.0 (8.0–34.0)	0.168

SD, standard deviation; BMI, body mass index; JAK, Janus kinase; HLA-B27, antigen B27; CRP, C-reactive protein; IQR, interquartile range; ESR, erythrocyte sedimentation rate; NSAID, non-steroidal anti-inflammatory drug.

### 3.2 Back pain and morning stiffness

**FIGURE 1:**
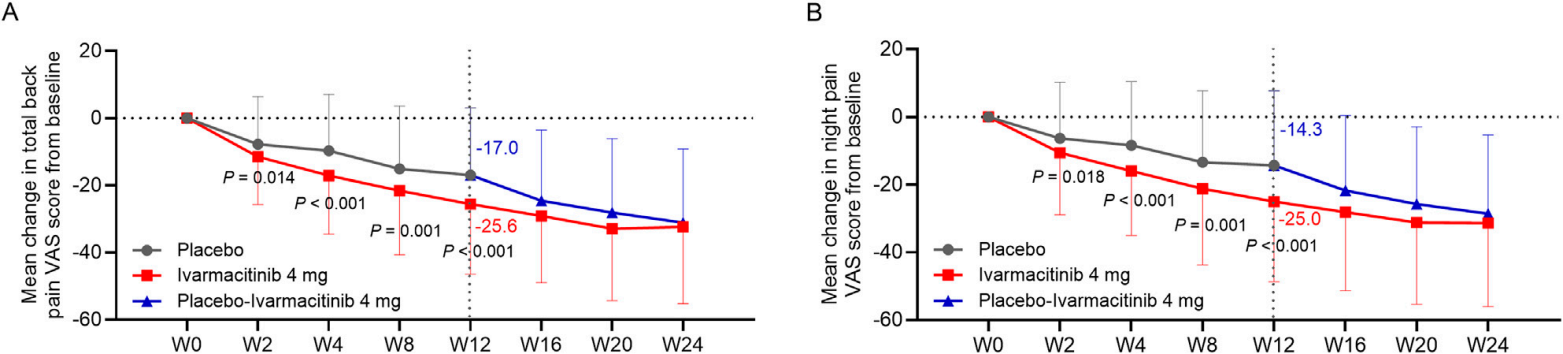
Total back pain and night pain. The mean change in total back pain **(A)** and night pain **(B)** VAS scores from baseline in ivarmacitinib and placebo/placebo-ivarmacitinib groups.

**FIGURE 2:**
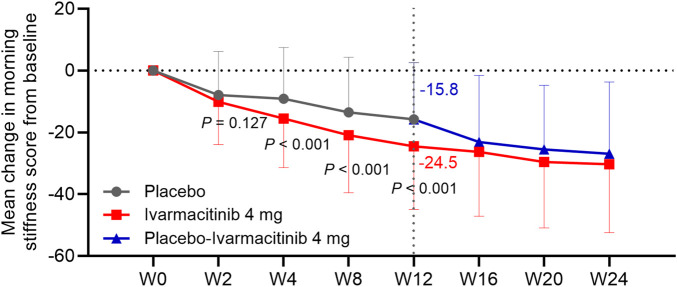
Morning stiffness. The mean change in morning stiffness score from baseline in ivarmacitinib and placebo/placebo-ivarmacitinib groups.

At W2, W4, W8, and W12, ivarmacitinib group showed significantly greater reductions in total back pain (−25.6 vs. −17.0 at W12) and night pain VAS (−25.0 vs. −14.3 at W12) scores from baseline compared with the placebo group (all *P* < 0.05, Figures 1A,B). Meanwhile, at W4, W8, and W12, the reductions in morning stiffness score from baseline were more profound in the ivarmacitinib group than in the placebo (−24.5 vs. −15.8 at W12; all *P* < 0.001, Figure 2). From W12 to W24, the ivarmacitinib group achieved sustained improvements in total back pain and night pain VAS scores and morning stiffness score. Moreover, the placebo-ivarmacitinib group (patients who switched from placebo to ivarmacitinib 4 mg at W12) demonstrated substantial enhancements in these outcomes during W12 to W24 (Figures 1, 2).

### 3.3 Global assessment and ASQoL

**FIGURE 3:**
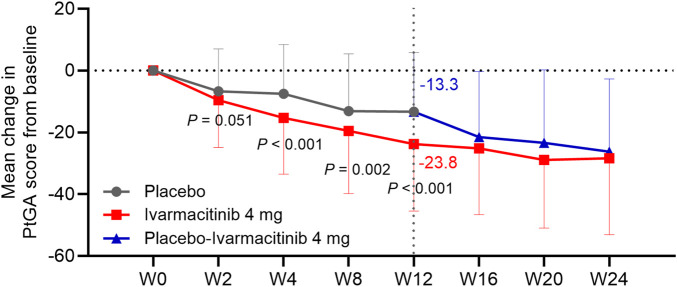
PtGA. The mean change in PtGA score from baseline in ivarmacitinib and placebo/placebo-ivarmacitinib groups.

**FIGURE 4:**
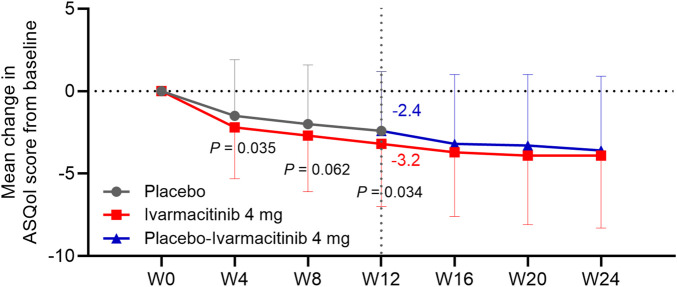
ASQol. The mean change in ASQol score from baseline in ivarmacitinib and placebo/placebo-ivarmacitinib groups.

At W4, W8, and W12, the ivarmacitinib group showed significantly greater reductions in PtGA score from baseline compared with the placebo (−23.8 vs. −13.3 at W12; all *P* < 0.01). The reduction in PtGA score from baseline to W2 was also greater in the ivarmacitinib group than in the placebo group, but did not achieve statistical significance (*P* = 0.051, Figure 3). Except at W8, improvements in ASQoL score from baseline at each time point up to W12 were more substantial in the ivarmacitinib group compared to the placebo group (−3.2 vs. −2.4 at W12; all *P* < 0.05, except for W8, Figure 4). During W12 to W24, the improvements in PtGA and ASQol scores were sustained in the ivarmacitinib group and were noted in the placebo-ivarmacitinib group (Figures 3, 4).

### 3.4 SF-36

**FIGURE 5:**
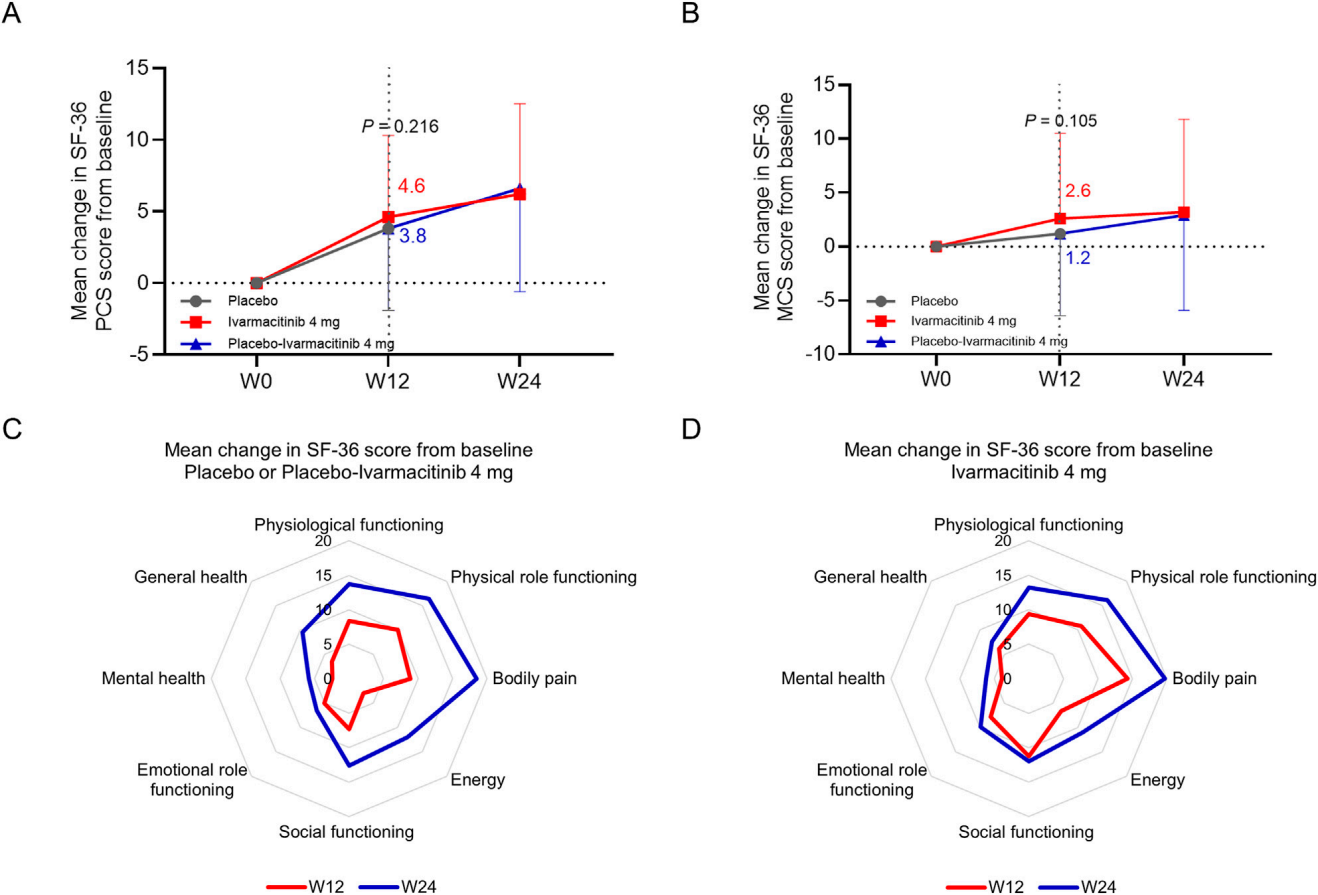
SF-36 PCS and MCS. The mean change in SF-36 PCS **(A)** and MCS **(B)** scores from baseline in ivarmacitinib and placebo/placebo-ivarmacitinib groups. The mean change in different dimensions of SF-36 from baseline in placebo/placebo-ivarmacitinib **(C)** and ivarmacitinib **(D)** groups.

At W12, the changes in SF-36 physical component score (PCS) (4.6 vs. 3.8, *P* = 0.216, Figure 5A) and mental component score (MCS) (2.6 vs. 1.2, *P* = 0.105, Figure 5B) from baseline were numerically higher in the ivarmacitinib group compared with the placebo group, but did not achieve statistical significance. At W24, both ivarmacitinib and placebo-ivarmacitinib groups achieved substantial improvements in SF-36 PCS and MCS scores.

At W12, placebo group exhibited limited improvements in SF-36 PCS and MCS dimensions; while the improvements in almost all dimensions were relatively profound in the ivarmacitinib group. At W24, almost all dimensions exhibited profound improvements in placebo-ivarmacitinib group, and were further improved in the ivarmacitinib group (Figures 5C,D).

### 3.5 Patient-reported disease activity and functioning

**FIGURE 6:**
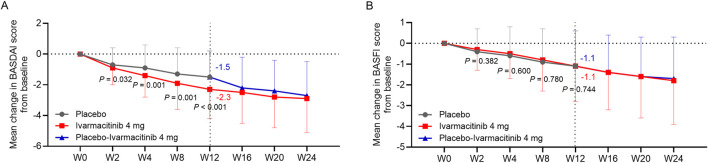
BASDAI and BASFI. The mean change in BASDAI **(A)** and BASFI **(B)** scores from baseline in ivarmacitinib and placebo/placebo-ivarmacitinib groups.

Ivarmacitinib group exhibited greater reductions in BASDAI score from baseline compared with the placebo group at W2, W4, W8, and W12 (−2.3 vs. −1.5 at W12; all *P* < 0.05, Figure 6A). BASFI score was continuously improved in both groups, while no statistical difference was observed between groups at each time point from W0 to W12 (−1.1 vs. −1.1 at W12; all *P* > 0.05, Figure 6B). During W12 to W24, both BASDAI and BASFI scores were continuously improved in ivarmacitinib and placebo-ivarmacitinib group (Figures 6A,B).

## 4 Discussion

In the phase II/III trial, the ASAS20 and ASAS40 response rates at W12 were 48.7% and 32.1% in the ivarmacitinib group compared with 29.0% and 18.3% in the placebo group, suggesting the efficacy of ivarmacitinib in patients with active AS (Liu et al., 2025c). Considering that improving the health-related QoL and daily functioning is the ultimate treatment goal for AS, the assessment of PROs is critical (Agrawal et al., 2024). The current *post hoc* analysis used the data from the phase II/III trial; and reported several interesting findings (Liu et al., 2025c). Compared with placebo, ivarmacitinib 4 mg demonstrated a superior effect on promoting different dimensions of PROs during 12 weeks of treatment. Meanwhile, ivarmacitinib 4 mg provided sustained benefits on these PROs through 24 weeks of treatment. Moreover, switching from placebo to ivarmacitinib 4 mg exerted notable promotions on PROs in patients with active AS.

A previous phase II/III trial demonstrated that after 12 weeks of treatment, ivarmacitinib 4 mg provided greater improvements in PROs compared with the placebo, providing a general view on the impact of ivarmacitinib on PROs in patients with active AS (Liu et al., 2025c). This *post hoc* analysis further investigated the data on PROs and found that during 12 weeks of treatment, improvements in most of the PROs were more notable by ivarmacitinib 4 mg compared with placebo. These PROs covered back pain, morning stiffness, health-related QoL, patient-reported disease activity, and patient global assessment, among which back pain VAS score represents pain, one of the core symptoms of AS (Nhan and Caplan, 2016). Moreover, the improvements in some of the PROs, such as ASQol, pain VAS, and BASDAI, were greater than the minimal clinically important improvements reported by previous studies (Gandomi et al., 2022; Karmacharya et al., 2023; Kvamme et al., 2010). These findings suggested that ivarmacitinib 4 mg exerted superior effects on PROs than placebo and provided clinically meaningful improvements in different PROs. These findings may be explained by its function as a selective JAK1 inhibitor. ivarmacitinib effectively suppressed the downstream cytokine signaling of JAK/STAT pathway, such as the interleukin-23/-17 axis, thereby reducing inflammation and alleviating AS symptoms (Ahmed et al., 2024; Schwartz et al., 2017; Smith and Colbert, 2014). In addition, the higher ASAS20 and ASAS40 response rates after ivarmacitinib treatment in patients with AS could also explain the improved PROs, which was in line with a previous study (Liu et al., 2025a). The phase II/III trial reported an ASAS20 of 48.7% in ivarmacitinib group compared with 29.0% in the placebo group (Liu et al., 2025c). Moreover, the superior improvements in back pain VAS score, night pain VAS score, and BASDAI score in the ivarmacitinib group were seen as early as W2, and an early improvement in these PROs was critical to promote the overall daily life in patients with active AS. Compared with other established treatments for AS such as tumor necrosis factor-α inhibitors and other JAK inhibitors, ivarmacitinib presented similar improvements in patient-reported disease activity and pain, suggesting that ivarmacitinib fits the current therapeutic landscape for AS (Baraliakos et al., 2023; Dong et al., 2023; Strand et al., 2019). Besides, according to a previous study, PROs showed linear associations with work productivity domains in patients with AS (Magrey et al., 2024). Therefore, it could be assumed that ivarmacitinib might also promote work productivity in patients with AS through improving PROs.

However, we also noticed that the changes in BASFI score from baseline were not different between ivarmacitinib and placebo during 12 weeks of treatment. The possible explanation is that: the items of BASFI questionnaire mainly focus on the daily functioning, such as standing without support and climbing steps without aid (Zochling, 2011). The intervention time might not be long enough to observe the difference in these functioning. Meanwhile, the change in SF-36 PCS score from baseline to 12 weeks of treatment was not different between groups, either. From the figures presenting the detailed dimensions of SF-36 (Figures 5C,D), some of the items related to the PCS score, such as physical functioning and physical role functioning, did not show notable difference between ivarmacitinib and placebo. This could also be due to scenario similar to that observed for the BASFI score (Lins and Carvalho, 2016). Moreover, the dimension of bodily pain was significantly improved by ivarmacitinib, suggesting the outstanding effect of ivarmacitinib on alleviating pain in patients with active AS. The lack of significance in SF-36 MCS could be also explained by the low intervention period.

In the phase II/III trial of ivarmacitinib in active AS, patients with placebo switched to ivarmacitinib 4 mg after 12 weeks of treatment, while those with ivarmacitinib 4 mg continued the regimen until 24 weeks (Liu et al., 2025c). In the current *post hoc* study, all PROs were continuously improved until 24 weeks of treatment in patients receiving ivarmacitinib 4 mg, which highlighted the consistent effect of ivarmacitinib in improving various dimensions of PROs in patients with active AS. Moreover, in patients who switched from placebo to ivarmacitinib 4 mg, notable improvements in all PROs were observed. These findings suggested that patients who delayed the initiation of ivarmacitinib could also experience meaningful improvements in PROs.

The impact of ivarmacitinib in improving PROs in patients with active AS could be supported by previous studies (McInnes et al., 2022; Navarro-Compán et al., 2023; Navarro-Compán et al., 2022) For example, a *post hoc* study of a phase III clinical trial reported that the twice daily administration of tofacitinib 5 mg improved BASDAI spinal pain, BASDAI fatigue, ASQol, and work impairment after 16 weeks of treatment compared with placebo in patients with active AS (Navarro-Compán et al., 2022). A *post hoc* study of three clinical trials showed that upadacitinib 15 mg daily reduced pain compared with placebo and sustained after 1 year of treatment in patients with active AS (McInnes et al., 2022). Moreover, a *post hoc* study of a phase III trial revealed that upadacitinib improved various PROs reflecting back pain, fatigue, patient-reported disease activity, and QoL in patients with active AS who were unresponsive to biologics (Navarro-Compán et al., 2023). The findings of our study were partly similar to those of previous studies.

The differences in the PROs in this *post hoc* study and the phase II/III trial could be explained by different statistical methods (observed means vs. least squares means). Meanwhile, this *post hoc* study had several limitations. Firstly, limited by the treatment duration of the phase II/III trial, this *post hoc* study only evaluated the effect of ivarmacitinib on PROs during 24 weeks of treatment. However, considering that AS is a chronic disease that requires sustained treatment, the long-term PROs after ivarmacitinib treatment under real-world settings should be further explored. Secondly, only patients from China were included in the trial, which hindered the generalizability of our findings. Thirdly, the phase II/III trial lacked an active comparator. Therefore, the effect of ivarmacitinib on PROs compared to other treatments for AS, such as tumor necrosis factor inhibitors or interleukin-17 inhibitors, should be further investigated. Fourthly, questionnaires for fatigue were not assessed after treatment of ivarmacitinib, which should be addresses in future studies.

## 5 Conclusion

Conclusively, ivarmacitinib improves multiple dimensions of PROs in patients with active AS, supporting its clinical application. Switching to ivarmacitinib also provides substantial benefits in diverse PROs, ensuring that patients who delay the initiation of ivarmacitinib also experience meaningful improvements in PROs.

## Data Availability

The original contributions presented in the study are included in the article/supplementary material, further inquiries can be directed to the corresponding author.
